# The three principles of action: a Pavlovian-instrumental transfer hypothesis

**DOI:** 10.3389/fnbeh.2013.00153

**Published:** 2013-11-19

**Authors:** Emilio Cartoni, Stefano Puglisi-Allegra, Gianluca Baldassarre

**Affiliations:** ^1^Laboratory of Computational Embodied Neuroscience, Istituto di Scienze e Tecnologie della Cognizione, Consiglio Nazionale delle RicercheRome, Italy; ^2^Dipartimento di Psicologia and Centro Daniel Bovet, Sapienza Università di RomaRome, Italy; ^3^Fondazione Santa Lucia, IRCCSRome, Italy

**Keywords:** Pavlovian-instrumental transfer, specific PIT, general PIT, goal-directed behavior, Bayesian network, latent causes, nucleus accumbens

## Abstract

Pavlovian conditioned stimuli can influence instrumental responding, an effect called Pavlovian-instrumental transfer (PIT). During the last decade, PIT has been subdivided into two types: specific PIT and general PIT, each having its own neural substrates. Specific PIT happens when a conditioned stimulus (CS) associated with a reward enhances an instrumental response directed to the same reward. Under general PIT, instead, the CS enhances a response directed to a different reward. While important progress has been made into identifying the neural substrates, the function of specific and general PIT and how they interact with instrumental responses are still not clear. In the experimental paradigm that distinguishes specific and general PIT an effect of PIT inhibition has also been observed and is waiting for an explanation. Here we propose an hypothesis that links these three PIT effects (specific PIT, general PIT and PIT inhibition) to three aspects of action evaluation. These three aspects, which we call “principles of action”, are: context, efficacy, and utility. In *goal-directed* behavior, an agent has to evaluate if the context is suitable to accomplish the goal, the efficacy of his action in getting the goal, and the utility of the goal itself: we suggest that each of the three PIT effects is related to one of these aspects of action evaluation. In particular, we link specific PIT with the estimation of efficacy, general PIT with the evaluation of utility, and PIT inhibition with the adequacy of context. We also provide a latent cause Bayesian computational model that exemplifies this hypothesis. This hypothesis and the model provide a new framework and new predictions to advance knowledge about PIT functioning and its role in animal adaptation.

## 1. Introduction

Pavlovian conditioned stimuli (CS) associated to a reward can affect instrumental responding toward the same or a different reward. This effect is called *Pavlovian-instrumental transfer* (PIT). As an example, in a typical experimental scenario a rat is trained to associate a sound (CS) with the delivery of food. Later, the rat undergoes an instrumental training where it learns to press a lever to get some food (without the sound being present). Finally, the rat is presented again with the opportunity to press the lever, this time both in the presence and absence of the sound. The results show that the rat will press the lever more in the presence of the sound than without, even if the sound has not been previously paired with lever pressing. The Pavlovian sound-food association learned in the first phase has somehow transferred to the instrumental situation, hence the name “Pavlovian-instrumental transfer.”

In recent years, this effect has been further subdivided into specific and general PIT. Specific PIT happens when the CS is paired with the same reward of the instrumental action. Instead, general PIT happens when the CS is paired with a different reward. In both cases, the presence of the CS leads to higher instrumental responding, however, different neural substrates are involved. Specific PIT involves the *basolateral amygdala* and the *nucleus accumbens shell*. General PIT involves *central amygdala* and the *nucleus accumbens core* (Corbit and Balleine, 2005, 2011). While most of the Pavlovian-instrumental transfer experiments have been done with rats, PIT is also present in humans and seems to involve the same brain structures (Prévost et al., 2012). Despite these advances in associating PIT effects to specific brain areas, the specific neural mechanisms underlying them are still unknown.

At the functional level, the picture is not fully clear either. Both Pavlovian and instrumental learning are often thought about in associationist terms. In associationist terms, Pavlovian conditioning leads to learning stimulus-outcome associations while instrumental conditioning can lead to associations between responses and their outcomes. One straightforward way of explainining PIT could be then in terms of a stimulus-outcome-response (S-O-R) chain. According to this view, during Pavlovian learning the subject learns a stimulus-outcome association (S-O); while during instrumental training it learns both a R-O (response-outcome) association and its inverse O-R (outcome-response) association. In the PIT test phase, hearing the sound (S) triggers the activation of the food outcome representation (O) thanks to the S-O association; this representation in turn activates its associated response through the O-R association, thus increasing instrumental responding. In general PIT, however, the outcome in the S-O association is not the same outcome of the O-R association. This case is thus explained by referring to the general motivating properties of a rewarding outcome instead of its specific sensory properties, so that the CS presence can still enhance instrumental responding, even if the CS is associated to a different reward. However, the S-O-R chain and the general motivation explanations leave some issues unresolved. For example, they do not explain why there is no general PIT effect when the conditioned stimulus (CS) is associated with a reward given by a different instrumental response than the one currently available (see paradigm in section 2). In this case, one would expect that, as the CS-evoked reward is different compared to the one currently available through instrumental action, a non-specific (general) PIT effect should happen. The absence of any enhancing PIT effect in this particular condition is currently attributed to a non-well defined inhibitory effect (Corbit and Balleine, 2005, 2011). Moreover, the S-O-R chain and general motivation explanations indicate *what* “computation” the agent is doing but they do not say *why* the agent is doing it.

Our proposal is that, in *goal-directed behavior*, each of the three PIT processes (specific PIT, general PIT and PIT inhibition) plays a role in the evaluation of different aspects of the action (*principles of action*). In particular, we posit that an agent that wishes to act to accomplish a goal needs to take into account at least three aspects: context, efficacy and utility. The principle of *context* means that an action needs the right context to reach its goal—e.g., it is useless to call for a waiter if you are not at a restaurant. *Efficacy* is the probability of reaching a goal: not all actions are guaranteed to accomplish a goal. For example, buying a single lottery ticket has few chances of success. *Utility* means that the result of the action can be more or less valuable, depending on the state of the agent. For example, pressing a lever for food has a high value if the animal is hungry, less or no value if it is sated. According to our hypothesis, an animal considers all these three aspects when it chooses which action to perform. The three PIT effects (specific PIT, general PIT and PIT inhibition) are each related to one of these aspects. We will link specific PIT with efficacy (chances of success), general PIT with utility (value of the future state) and PIT inhibition with context (availability of certain rewards).

We will also propose a Bayesian computational model that exemplifies our hypothesis. The model will build upon previous work in the Pavlovian literature that conceptualizes Pavlovian conditioning as latent-causes learning. The interplay of latent-causes (which can also be thought as contexts) will affect the number of available rewards and their probability, capturing the three PIT processes as in our hypothesis.

Our hypothesis and the model together provide a new framework and new predictions to advance knowledge about PIT. Our proposal is strongly tied to *goal-directed* behavior. Indeed we think that it is important to study PIT because if offers a peculiar window of observation on both Pavlovian and instrumental processes. Hopefully future work on this line of research will improve not only our understanding of PIT but of Pavlovian and instrumental processes as well.

In the following sections, we will first review the current experimental paradigm used to distinguish specific and general PIT (section 2). We will then discuss some issues with current explanations of PIT (section 3). After that, we will explain our hypothesis (section 4) and provide a computational model for it (section 5). In the final section we will discuss new predictions and limitations of the hypothesis and draw some final conclusions (section 6).

## 2. PIT paradigm

**Figure 1:**
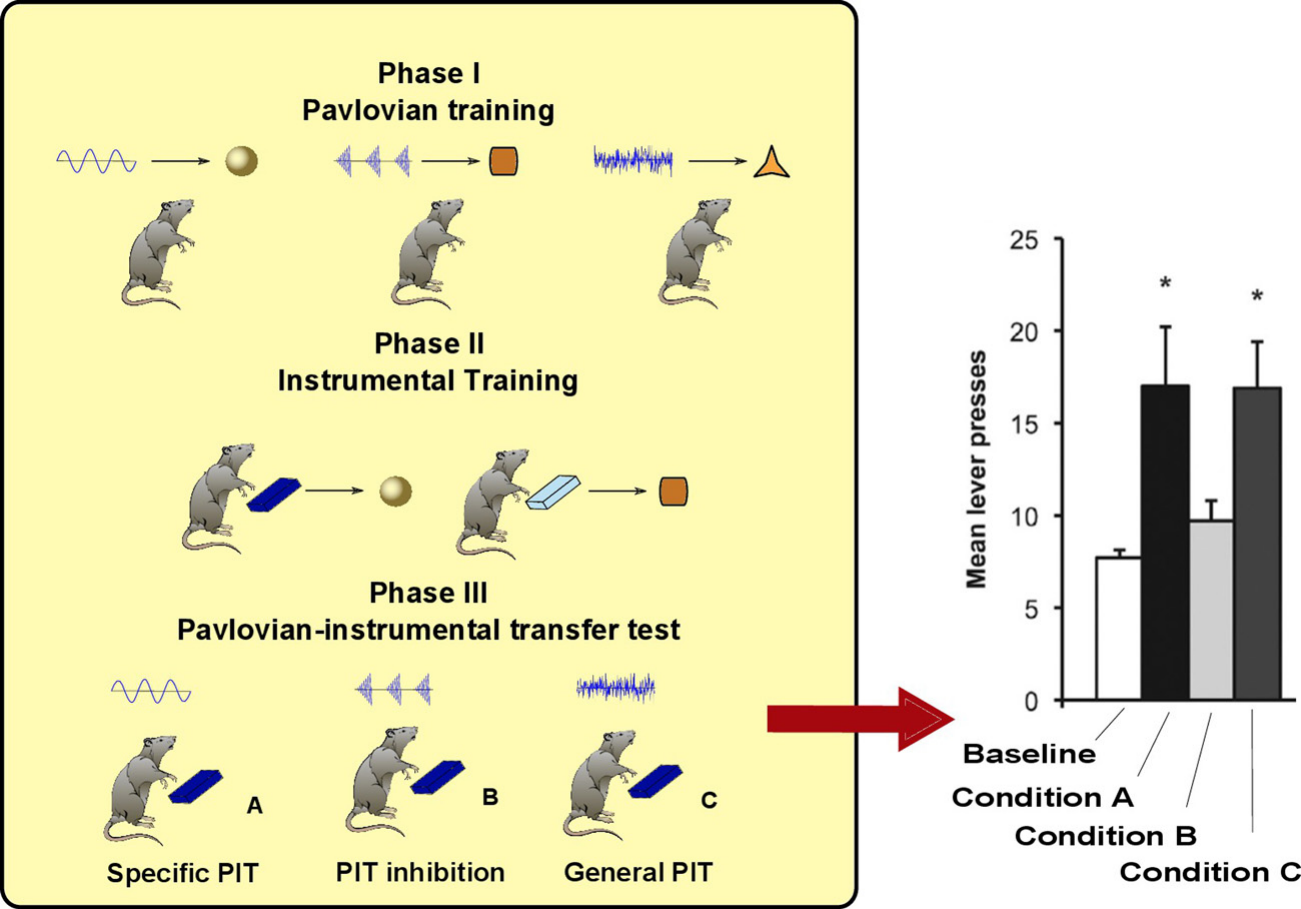
**Left:** PIT paradigm to distinguish specific and general PIT. **Right:** histogram showing typical results of the test phase, in terms of lever press frequency. ^*^Responding is significantly higher in Condition A and C. Histogram adapted with permission of Society for Neuroscience (Corbit and Balleine, 2011).

As noted in the introduction, PIT is not a unitary process. The experimental paradigm to distinguish specific PIT and general PIT was introduced by Corbit and Balleine (2005) (see Figure 1). It involves three phases: a Pavlovian phase, an instrumental phase and a test phase. In the Pavlovian phase, three sounds are associated with delivery of three different foods. In the instrumental phase, the rat undergoes two separate trainings: in each of these two trainings it learns to press a lever for a different food. The rewards used for the instrumental phase are two of the foods previously used in the Pavlovian training. A non-continuous reinforcement schedule is used, so the relationship between pressing the lever and obtaining food is probabilistic. For example, if a random-ratio RR20 schedule is used, the rat will get the reward about once every 20 lever presses. In the final phase PIT is tested. The rat is presented with one of the levers and each of the three sounds are played separated by some interval. This phase is done in extinction (no delivery of food) to prevent further reinforcement learning that could confound the results. When there is no sound, the rat will press the lever with a certain frequency (baseline). During two of the three sounds it will press the lever more than the baseline (PIT effect). Specifically, it will press more when it hears either the sound associated with the same reward of the lever (specific PIT) or the sound associated with a reward not used in the instrumental phase (general PIT). However, the sound *associated with the reward of the other lever* will not augment instrumental responding to the tested lever. Something is preventing PIT to be expressed in this latter case: we will call this effect *PIT inhibition* (we will return on this point in section 3).

**Figure 2:**
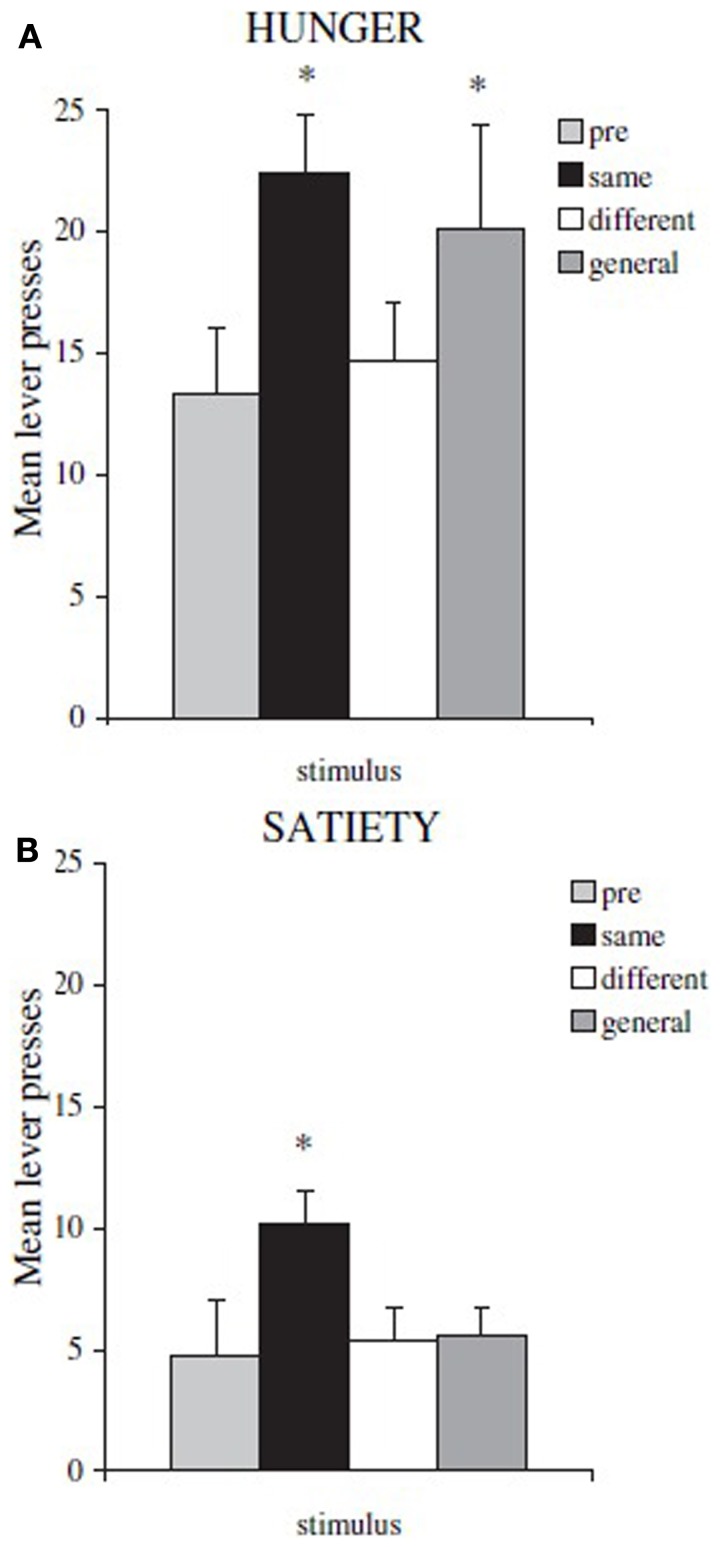
**Mean lever presses during PIT test phase, in hungry rats and in sated ones**. Shifting the motivational state from hunger to satiety (food devaluation) causes a general drop in instrumental performance and the disappearance of general PIT. **(A)** rats tested in hunger state: ^*^both same (specific PIT) and general (general PIT) conditions are higher than baseline **(B)** rats tested in sated state: ^*^only same (specific PIT) condition is higher than baseline. Reprinted with permission © The Authors (2007). Journal Compilation © Federation of European Neuroscience Societies and Blackwell Publishing Ltd., (Corbit et al., 2007). ^*^p <0.05.

By using this paradigm, Balleine and collegues have been able to identify the different neural substrates underlying specific and general PIT. Lesions to *nucleus accumbens shell* or *basolateral amygdala* eliminate specific PIT, while lesions to *nucleus accumbens core* or *central amygdala* eliminate general PIT (Corbit and Balleine, 2005, 2011). These substrates have also been confirmed by using inactivation and disconnection procedures (Shiflett and Balleine, 2010; Corbit and Balleine, 2011). Moreover, by using this paradigm a further important difference between specific and general PIT has been found: general PIT is subject to devaluation, while specific PIT is not. To show this, Corbit et al. (2007) executed the test phase of the paradigm after sating rats and they found that specific PIT was still present while general PIT disappeared (Figure 2). That is, general PIT is affected by the devaluation of food by satiation, while specific PIT is not. There have been previous reports about PIT being unaffected by devaluation but a different paradigm was used and no lesions were performed, so the type of PIT evoked (specific or general) in those experiments can only be inferred (Rescorla, 1994; Holland, 2004). In Holland (2004) some of the reported results can be interpreted as specific and general PIT being both unaffected by devaluation, in contrast with results from Corbit et al. (2007). We will discuss this contradiction in the final section.

## 3. Explaining PIT: current issues

As mentioned in the introduction, specific PIT can be thought in terms of a S-O-R chain: the sound stimulus evokes an outcome (food) and that food in turn evokes the associated response (pressing the lever). In the case of general PIT, instead, the sound evokes food and the reward of food exerts a general motivational effect on instrumental responses. As an example of this kind of explanations, we will look at the associative-cybernetic model of Balleine & Dickinson as reported by Ostlund and Balleine (2007). This is, to our knowledge, the most complete model in the PIT literature. The associative-cybernetic model is actually not just a model of PIT, but a more general model of both Pavlovian and instrumental processes. However it does include a way to explain both specific and general PIT functioning.

**Figure 3:**
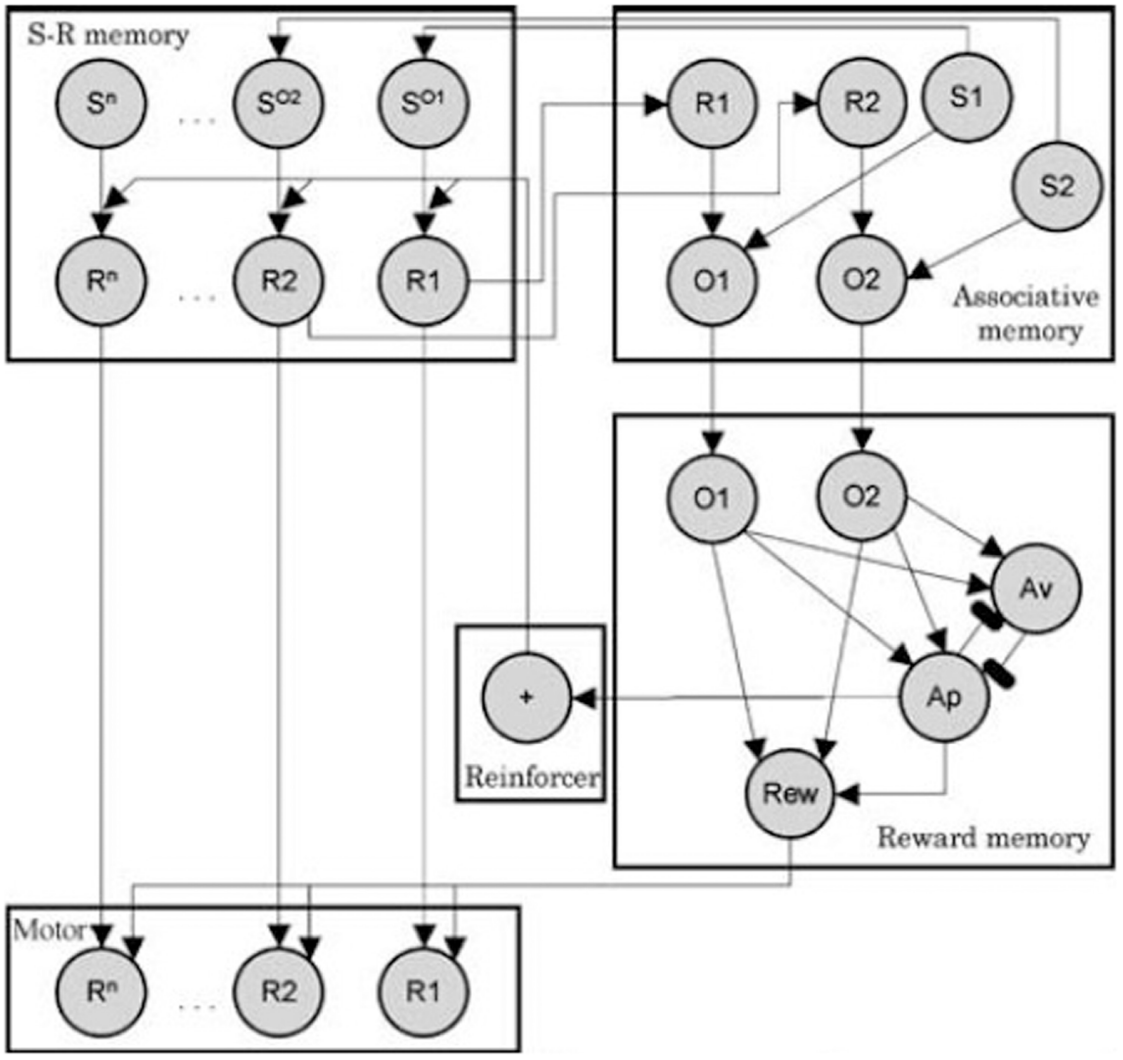
**Associative-cybernetic model by Balleine and Dickinson as reported by Balleine and Ostlund (2007)**. Reprinted with permission © 2007 New York Academy of Sciences.

In the model, Pavlovian stimulus-outcome associations are represented in the “associative memory” component (see Figure 3). According to Balleine and Ostlund (2007), during instrumental learning, both R-O and O-R association are learned. Indeed, food is not only the result of pressing the lever, but it also precedes the next lever pressing. So within the “S-R memory” component, food is considered as a stimulus (*S*^*O*^) that precedes the response and thus *S*^*O*^ − *R* associations are learned. When the Pavlovian sound stimulus is encountered, it activates its representation in the associative memory (e.g., *S*1). In turn, its representation activates its associatiated food outcome as a stimulus in S-R memory (*S*^*O*1^). This representation then activates the corresponding instrumental response (*R*1). This “pathway” in the model can thus explain how specific PIT works and it is basically a S-O-R chain explanation.

However, there is a second pathway in the model through which Pavlovian stimuli can influence instrumental responses. Assume that the rat now hears a different sound *S*2 associated with a different food outcome *O*2. The specific PIT pathway will not work because this food is not associated to *R*1 in the S-R memory. However, the sound *S*2 activates *O*2 in the associative memory. This activates the corresponding *O*2 in the “reward memory” component, which in turn activates the reward node (Rew). We can think the Rew node as an “expectation of value”. This expectation of value (Rew) can exert an excitatory effect on all motor responses (arrows from Rew to all responses *R*1..*R*^n^). Thus the sound stimulus can evoke an expectation of value through which it can aspecifically enhance an instrumental response associated with a different food. This effect can be assumed to be a model of general PIT. The fact that general PIT works through an expectation of value is also consistent with the fact that general PIT (and not specific PIT) is sensitive to devaluation.

**Figure 4:**
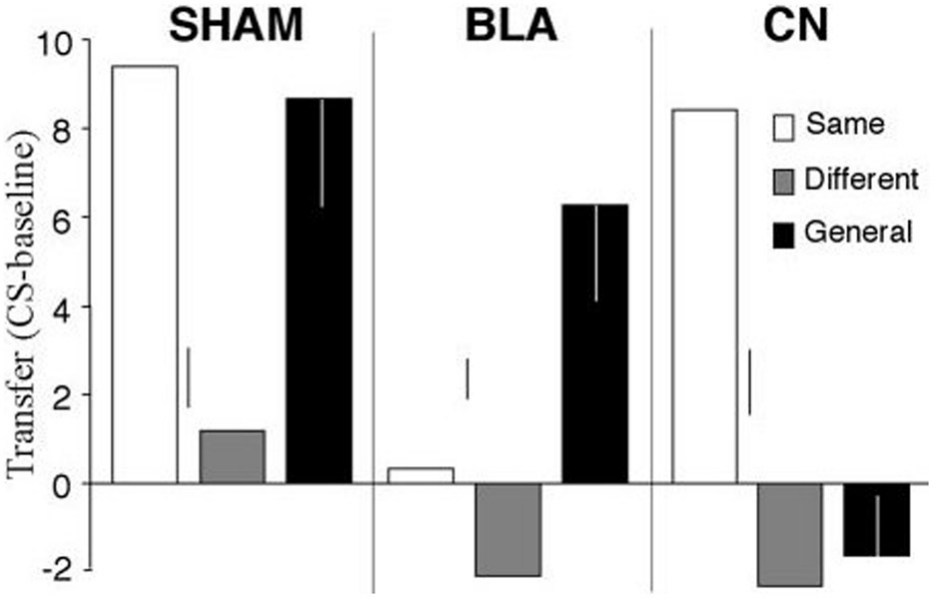
**PIT effects after lesions of basolateral amygdala (BLA) and central nucleus of amygdala (CN), compared to a sham lesion group**. The histogram shows PIT effects as frequency of lever pressing in the presence of CS minus lever frequency in the absence of CS (*baseline*). The control group (*sham*) shows specific PIT effect in the *same* condition (CS associated with the same reward as the instrumental response) and general PIT effect in the *general* condition (CS associated with a reward not used in the instrumental phase). Notice the absence of any PIT effect in the *different* condition where the CS is associated to the reward of the lever not available during the test. BLA and CN lesions eliminate specific PIT and general PIT, respectively. Reprinted with permission of Society for Neuroscience (Corbit and Balleine, 2005).

Even though the associative-cybernetic model can explain the existence of both specific and general PIT and their interaction with devaluation, some important aspects in PIT experimental data remain without answer and suggest that PIT phenomena need a more complex explanation. A first important aspect involves the absence of general PIT in the *same* condition. In the *same* condition, a CS associated with a certain food enhances an instrumental response directed to the same food. One might expect that, according to the associative-cybernetic model, the CS would also elicit general PIT, using both pathways at the same time. That is, the CS could elicit PIT both through the S1-SO1-R1 pathway and through the S1-O1-O1-Rew-R1 pathway (Figure 3). In other words, one might expect that the CS can have both a specific PIT effect, as it evokes the food associated with the instrumental response, and a general PIT effect as it evokes an expectation of value (food) that can motivate instrumental responses aspecifically. Experimental data shows that this is not the case. Figure 4 shows that BLA lesions, belonging to specific PIT circuit, eliminate all PIT effect in the *same* condition. Morever, lesion of central amygdala, which belongs to the general PIT circuit, does not have any effect in the *same* condition. This means that in this condition only specific PIT is expressed and there is no general PIT.

**Figure 5:**
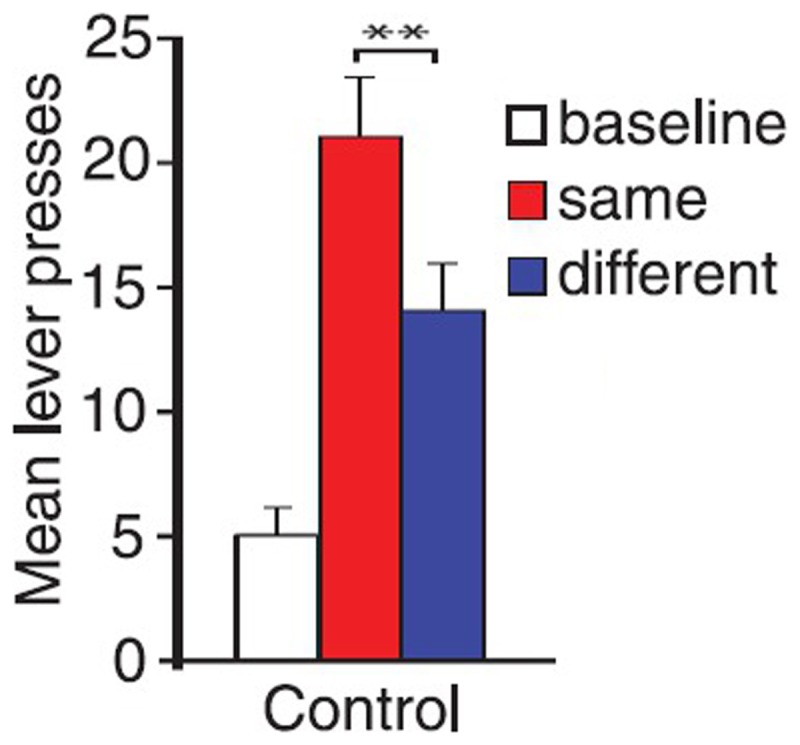
**PIT test phase in Corbit and Janak (2010)**. The control group in the figure displayed a noticeable PIT effect even in the *different* condition, albeit significantly less stronger than the one in the *same* condition (^**^). Experimental groups with dorsal striatum lesions (not shown here) also showed similar PIT effects in the *different* condition. Adapted with permission © The Authors (2010). Journal Compilation © Federation of European Neuroscience Societies and Blackwell Publishing Ltd., (Corbit and Janak, 2010). ^**^p < 0.01.

There is another case where the explanation of the associative-cybernetic model is incomplete. In the data shown in Figure 4 we can notice that in the *different* condition there is no PIT effect (neither specific nor general). The *different* condition corresponds to the case where the CS is associated with the reward of the other, not-available, instrumental response—that is, the reward of the lever not used during that test. One would expect that, as the CS is not associated to the same reward of the available instrumental response, it cannot elicit specific PIT but it would still elicit general PIT. On the contrary, there is no visibile PIT effect: the instrumental response stays at baseline level, even after lesions to the specific or general PIT circuit (see Figure 4). The associative-cybernetic model does not offer an explanation for this. There is no connection in the model that can explain why the aspecific effect of the “general PIT pathway” should be *inhibited* toward some instrumental responses. Even those articles that contain experimental data about this absence of general PIT simply suggest that there must be some kind of inhibitory effect (Corbit and Balleine, 2005), or they suggest possible neural locations of this inhibitory effect (Corbit and Balleine, 2011), but without exactly explaining its presence. So there is an inibitory effect, capable of suppressing general PIT, waiting to be explained. This effect is not always of the same entity: in some cases it is possible to have some kind of “partial” PIT effect in the *different* condition, as shown in Figure 5. This means that the inhibitory effect has not been able to completely suppress general PIT. We will return on this in the final section, suggesting one possible way on how it might happen.

**Figure 6:**
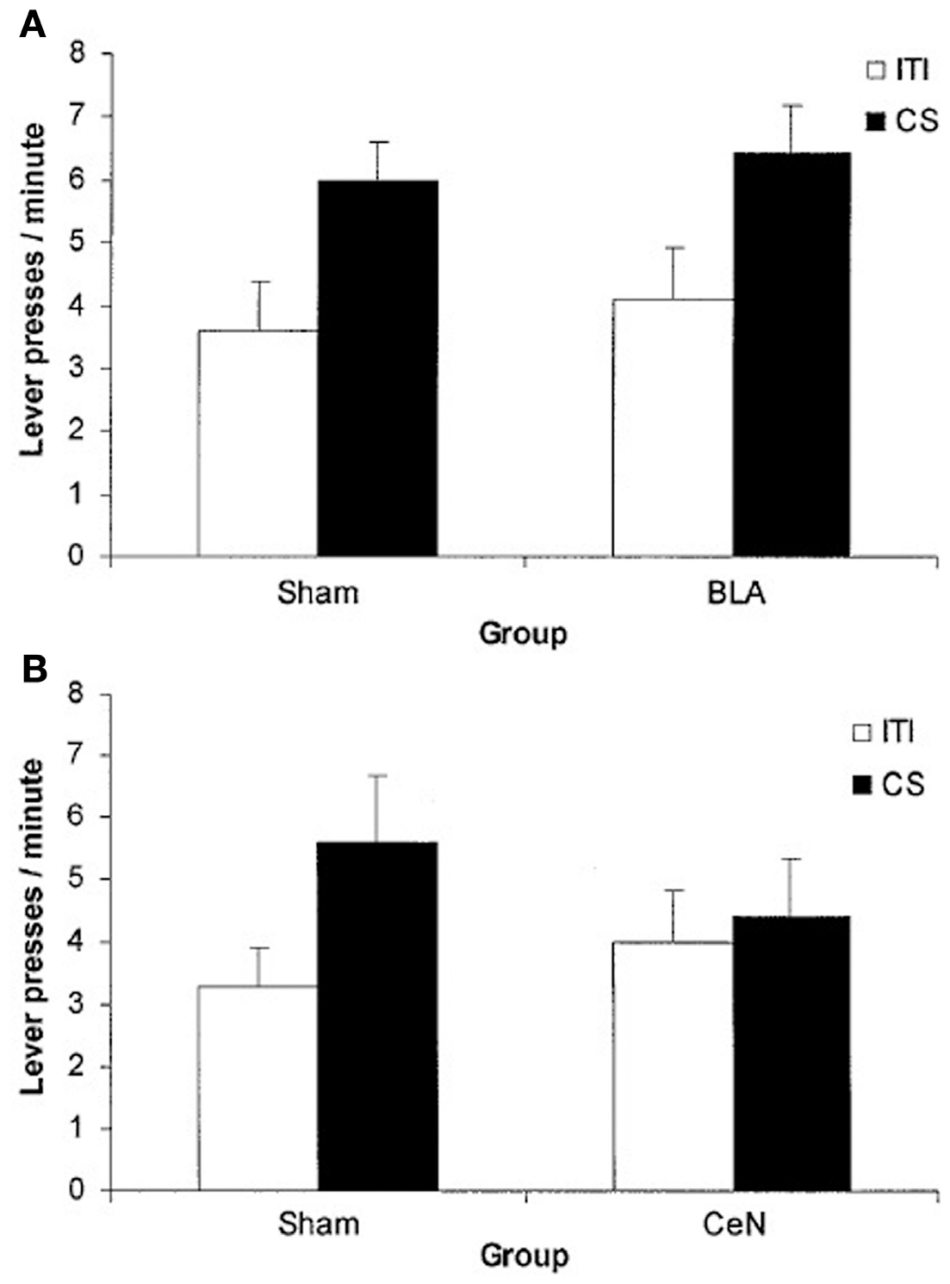
**Mean lever press during PIT test phase**. During training a single CS and a single lever were used, associated to the same food reward. The CS enhances instrumental response compared to baseline (ITI) only in the group with *basolateral amygdala* (BLA) lesion and in the control groups (Sham). Even though the same reward has been used for both the CS and the instrumental response, it is the *central amygdala* (CeN) lesion (general PIT circuit) to prevent PIT effect and not the one to the *basolateral amygdala* (specific PIT circuit). **(A)** comparison of BLA lesion group vs. Sham lesion group: both groups show a PIT effect during the CS period **(B)** comparison of CeN group vs. Sham lesion group: the CeN group instrumental response is not enhanced by the CS. Reprinted with permission © Federation of European Neuroscience Societies (Hall et al., 2001).

Finally, there is a third kind of data that asks for a different explanation than the two simple pathways. At the beginning of this century, a contradiction in the PIT literature arose. Blundell et al. (2001) reported that lesions to the basolateral amygdala affected PIT, while Hall et al. (2001) reported that lesions on central amygdala, and not basolateral amygdala, abolished PIT (see Figure 6). This contradiction was then resolved in Corbit and Balleine (2005) by showing that there exist two kinds of PIT, one depending on basolateral amygdala (specific PIT) and one depending on central amygdala (general PIT). However, one question remained open: why did Hall et al. (2001) procedure elicit general PIT (as shown by the sensitivity to central amygdala) instead of specific PIT as Blundell et al. (2001)? Hall et al. (2001) used a single CS and a single instrumental response, both associated to the same reward. Blundell et al. (2001), instead, used two CS and two levers with two different rewards. In both cases an interval reinforcement schedule was used. Given that Hall et al. (2001) used the same food for both the CS and the lever, one would expect a specific PIT, not a general PIT. Later articles usually refer to that fact simply by saying that procedures with a single lever seem to elicit general PIT instead of specific PIT (Corbit and Balleine, 2005, 2011). Our view is that it could be not just a question of the number of levers, but of the kind of instrumental response elicited: *habitual* versus *goal-directed*. It is known that interval schedules with a single lever easily lead to *habitual* behavior, while single-lever random ratio schedules and two-levers procedures (even using interval schedules) usually elicit *goal-directed* behavior (Yin and Knowlton, 2006). We suggest that the real reason underlying the lack of specific PIT effect in Hall et al. (2001) is that the elicited behavior was *habitual*. Our hypothesis, illustrated in the next section, will link specific PIT with the probability of reaching the goal of an action. Since in *habitual* behavior the action is a simple “reaction” to a stimulus and there is no goal evaluation, specific PIT cannot happen; general PIT, instead, not being tied to the specific consequences of the action, might still happen. Thus, Hall et al. (2001), by eliciting habitual behavior, measured general PIT instead of specific PIT and found it to be sensitive to central amygdala lesions, whereas Blundell et al. (2001) observed specific PIT in goal-directed behavior and found it to be affected by basolateral amygdala lesions.

## 4. Three principles of action: context, efficacy, utility

Experimental data from the PIT test phase shows three different conditions with three different effects: specific PIT, general PIT and PIT inhibition. We will now explain our hypothesis according to which these three effects are functionally linked to three aspects of action evaluation during *goal-directed behavior*.

An instrumental action, such as lever pressing connected to food, can be either *habitual* or *goal-directed* (Yin and Knowlton, 2006). In the case of *habitual* behavior, the instrumental action is elicited by a simple stimulus-response association and there is no evaluation of the consequences. In the case of *goal-directed* behavior, instead, the response is directed to a goal and it is linked to the evaluation of its consequences. These two types of behavior can be distinguished by devaluation and contingency alteration procedures. In the devaluation procedure food is devalued (e.g., by satiety) while in the contigency alteration procedures the relationship between pressing the lever and the presence of food is altered (e.g., pressing the lever now stops food delivery). In the case of habitual behavior, the rat (or other subject) will keep pressing the lever, while in the case of goal-directed behavior it will stop pressing it when the ability of the action to obtain food or the value of available food are altered. That is, goal-directed behavior distinguishes itself because action consequences are evaluted, both in terms of probability of happening and value.

Our hypothesis is that, during *goal-directed* behavior, the three aspects of PIT (specific PIT, general PIT, PIT inhibition) are linked to three aspects of this evaluation.

- **Context**. A goal-directed action must be executed in the right context (it is useless to call for a waiter if you are not at the restaurant).
- **Efficacy**. A goal-directed action can be more or less effective in reaching its goal (buying a single lottery ticket has few chances of winning).
- **Utility**. The goal of an action can be more or less useful (getting food is useful if you are hungry, less or not useful if you are sated).

General PIT can be linked to the principle of *utility*: a CS associated with food evokes a reward in the near future. Our hypothesis is that this reward is added to the future scenario of the consequences of the action, thus enhancing the motivation to execute it. However, if the animal is sated, this added reward clearly has no longer any value, so the motivation effect vanishes. As we saw before, satiety does indeed eliminate general PIT (Corbit et al., 2007). Moreover, the motivational effect of general PIT is possible only if the reward evoked by the CS is an “additional reward” compared to the one already foreseen by the action. In agreement with this, data collected using *random-ratio* schedules (where the behavior is usually goal-directed) show that in the specific PIT condition (where the CS reward is the same as the lever) general PIT does not happen (Corbit and Balleine, 2005, 2011).

Specific PIT can be linked instead to the principle of *efficacy*. A CS associated to the same reward as the lever predicts that this reward will be present in the near future. During the action evaluation, the CS acts as a cue that there is an higher chance of getting the reward associated to the action, thus motivating the agent to pursue that action. As specific PIT is then about an *increase of probability* of getting the food, this effect is immune to devaluation: whether the animal is sated or not, the CS predicts an higher probabily of reward, so, compared to the CS absence, the action will be evalued more positively. In agreement with this link between specific PIT and probability, in a human study, Trick et al. (2011) found that more predictive conditioned stimuli (those with higher probability of reward) induce a stronger PIT effect.

Lastly, PIT inhibition can be linked to the principle of *context*. We suggest that the presence of the lever acts as a discriminatory stimulus that inhibits the reward of the absent lever. That is, the presence of the lever signals not only that its associated reward is available, but also that it is *not* possible to obtain the reward of the other lever. The general PIT effect would be then inhibited by the presence of the lever associated with a different reward compared to the CS.

In other words, specific PIT represents the ability of an agent to take into account cues that indicate that a certain reward is more probable in the environment compared to when those cues are absent. The fact that those rewards are more probable translates into a perceived *higher efficacy* of the action. General PIT represents instead the ability to use cues that indicate the presence of other “additional” rewards in the environment, thus motivating the agent to act as they constitute an added value (*utility*). Finally, PIT inhibition represents the ability to take into account the context and to inhibit rewards signals that are known to be not available through action at that time (*context*). In the following section, we will now try to exemplify our hypothesis through a computational model.

## 5. A bayesian model of PIT

Bayesian modeling is increasingly used in many fields, from chemistry (Hibbert and Armstrong, 2009) to astrophysics (Loredo, 1992), from economy (Poirier, 2006) to genetics (Beaumont and Rannala, 2004). This increasingly widespread use has even prompted some to call it a “Bayesian revolution” (Beaumont and Rannala, 2004). Bayesian approaches are now being used in cognitive science too: indeed, both Pavlovian (Courville et al., 2004, 2005, 2006; Gershman and Niv, 2012) and instrumental (Solway and Botvinick, 2012; Pezzulo et al., 2013) Bayesian network models have been created.

The Bayesian approach owes its name to the Bayes theorem:

(1) $$p(h|d)=\frac{p\left(d|h\right)p(h)}{p(d)}$$

The theorem says that the *posterior* probability of an event *h* given a set of observations *d*, denoted with *p*(*h*|*d*), is equal to the probability of observing *d* given the event *h*, denoted with *p*(*d*|*h*), multiplied by the *a priori* probability of *h*, divided by the *a priori* probability of observing *d*. In other words, the theorem transforms the *a priori* probability of *h* in a *posterior* probability of *h* that takes into account the set of observations *d*. Bayes theorem tells us how to “update” in an optimal way our belief about *h* happening given the data *d* at our disposal. This theorem can then be used to create models about how an animal can make sound inferences about the events of the world given what it sees. Indeed, we have chosen to use this approach to simulate Pavlovian and instrumental learning and so to build a Bayesian computational model of PIT.

In the Pavlovian literature, Pavlovian learning is often thought as S-O learning, that is, as the acquisition of the association between a stimulus (S) and an outcome (O). In particular, associationist models usually focus on the predictive properties of S. Models such as Rescorla-Wagner (Rescorla and Wagner, 1972) or Pearce (Pearce, 1994) try to explain how S comes to predict outcome O (and thus elicit a Pavlovian response). However, a different approach exists. For example, Courville et al. (2005) describes Pavlovian learning using a Bayesian generative model with hidden latent causes. In this model, the animal does not simply try to learn how often O occurs after S. Instead, the animal tries to learn the whole *generative model*: that is, it tries to learn the hidden cause that makes both S and O appear. Indeed, this is also a more rational strategy by the animal, as in Pavlovian experiments S does not really cause O, but the two simply occur together because of a common cause (the experimental setup). By using this model, Courville et al. explain phenomena that are otherwise not well accounted for by classical associationist models such as Rescorla-Wagner or Pearce ones (Courville et al., 2005).

**Figure 7:**
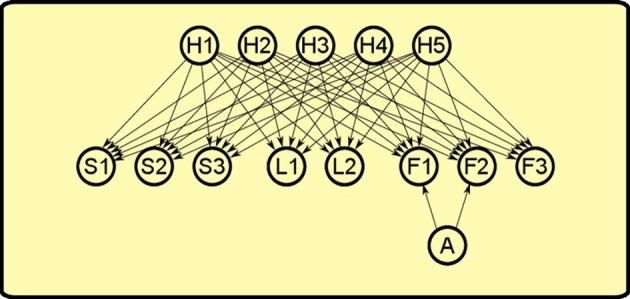
**Bayesian network used to simulate PIT**. There are three set of nodes: observable objects such as levers (L1, L2), sounds (S1, S2, S3) and foods (F1, F2, F3); latent causes that influence the presence of objects in the world (H1, H2, H3, H4, H5); the action of pressing the lever (A). Lever pressing is linked only to foods F1 and F2 as it can influence only their presence and not the other observables.

Inspired by this work, we “extended” it to the instrumental realm to explain PIT. As in Courville et al. (2005), our model is a *sigmoid belief network* (see Figure 7). The network is formed by three set of nodes:

- **Observables:** nodes that represent objects such as levers (L1, L2, L3), sounds (S1, S2, S3), foods (F1, F2, F3).
- **Latent causes:** nodes that represent hypothetic causes (H1, H2, H3, H4, H5) that can explain the observation or the lack of observation of the objects in the world.
- **Actions:** a single node that represents the action of pressing a lever (A) which can influence the presence of some objects in the world.

The activations of each node in the network represent the probability that the corresponding object is present. The nodes influence each other through their links. Each link is associated to a numerical *weight*. Those weights can be either positive or negative (or zero). Positive weights from a node to another mean that when the parent node is active, the child node is more likely to happen. Negative weights, instead, decrease the probability of the child node to be active. For simplicity, in this model only link weights are learned, while the number of hidden causes and their links are given. Learning link weights means that the agent has to discover how the hidden causes affect the probabilties of each observable and how its action can influence the presence of food. At first, we assigned a distribution of *a priori* probabilities to each link centered on the value of zero. This means that, before learning, the agent has not yet formed a particular “belief” on how these latent causes (or its action) can affect what he sees in the world. Then we trained the model by applying Bayesian inference on a set of simulated observations that represent Pavlovian and instrumental learning (following the paradigm described in section 2). As in Courville et al. (2005), we used a Monte Carlo Markov Chain method for training the model, in our case using WinBUGS software (Lunn et al., 2000). The resulting *a posteriori* distribution of probabilities describes the animal *belief* on how the world works after the conditioning sessions. The resulting model with weights based on the Pavlovian and instrumental training can account for the various PIT effects. We will now describe how the model behaves in each phase of the PIT paradigm and how it accounts for the three PIT effects: specific PIT, general PIT and PIT inhibition.

### 5.1. Pavlovian phase

**Figure 8:**
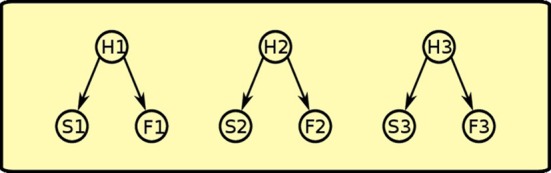
**Pavlovian phase: positive links between latent variables and co-occurring sounds and foods are established**.

During Pavlovian training, the rat sees that sound S1 and food F1 are correlated, so it assumes that an hidden cause H1 is generating both events. This happens for all the three Pavlovian trainings, thus generating positive weights between each of the three hidden causes (H1, H2, H3) and its pair of sound and food events (see Figure 8).

### 5.2. Instrumental phase

**Figure 9:**
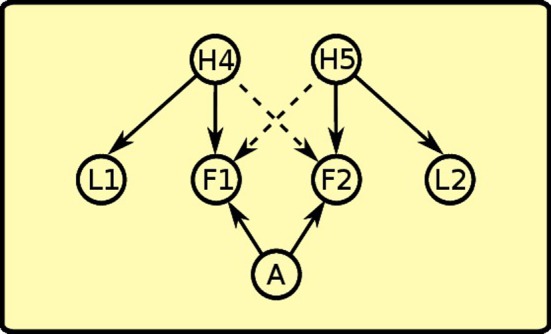
**Instrumental phase: instrumental latent causes, which give rise to the presence of the levers, interact with action to make food available**. Instrumental latent causes also inhibit each other's food availability (negative links depicted as dashed lines).

During instrumental learning, the rat sees that the presence of a lever L1 and food F1 availability are correlated, so it assigns a positive weight to the links between H4 and L1 and between H4 and F1 (see Figure 9). Food delivery, however, depends also on the action of lever pressing (A), so a positive link between action and food is also formed. The same happens for the other instrumental learning with lever L2 and food F2. The rat also learns a negative association (dashed line) between the “instrumental” hidden cause and the other food: in other words, with experience it discovers that when H4 is active and lever L1 is present, lever pressing (A) will not obtain food F2.

### 5.3. Test phase

**Figure 10:**
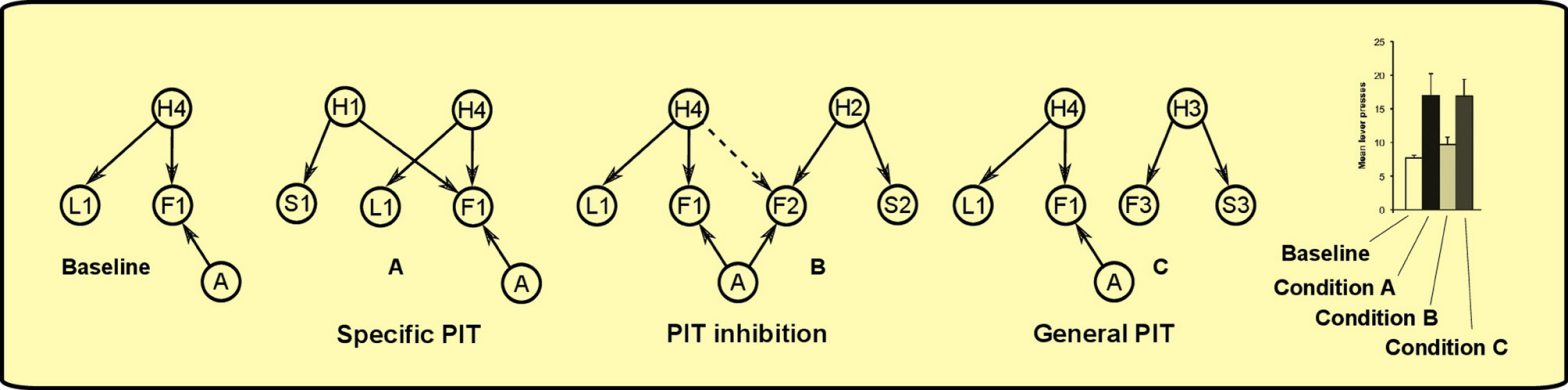
**PIT test phase**. Hearing different sounds gives rise to different interactions in the model. For clarity, only relevant nodes from the previous learning phases are shown in each condition. The model can match typical experimental results, such as the histogram from Corbit and Balleine (2011). Histogram adapted with permission of Society for Neuroscience.

The test phase involves four possible conditions depending on the presence of different sounds togheter with one of the levers. This will give rise to different patterns of activations in the trained model (see Figure 10):

**Baseline**: in the baseline condition, lever L1 is presented alone. The rat will press it with some frequency knowing, from previous instrumental learning, that when L1 is present it can get food F1 by lever pressing (action A).

**Condition A – specific PIT**: from the presence of S1 and L1, the rat can infer the presence of causes H1 and H4, which both predict F1 in the near future. This motivates the rat to press the lever more than when L1 is present alone, without any sound, as there are now increased chances of getting F1 in the immediate future.

**Condition C – general PIT**: the presence of lever L1 and sound S3 implies that causes H4 and H3 might be present and that foods F1 and F3 will appear in the future. While food F3 is not a direct effect of lever pressing A, its predicted presence nevertheless motivates the rat to press the lever more than the baseline condition. This is a different kind of motivation from Condition A: instead of augmenting the probablity of the food targeted by the action, it adds a new food reward to the scene.

**Condition B – PIT inhibition**: this condition is similar to condition C, but in this case food F2, evoked by sound S2 (through H2), is inhibited by cause H4. Thus, only food F1 remains predicted and no enhancement is found compared to the baseline.

The model thus exemplifies how our hypothesis could work. Specific PIT arises from the interaction between Pavlovian and instrumental latent causes that results in the evaluation of higher chances of getting the reward connected to the action (*principle of efficacy*). General PIT arises from adding value (a new reward) to the predicted future scenario (*principle of utility*). The absence of general PIT in the third condition could be instead consequence of the fact that the reward predicted by the CS is excluded as it is not possible in the presence of the particular lever (*principle of context*).

## 6. Conclusions

In this article, we have first reviewed some experimental data about Pavlovian-instrumental transfer. These data suggest that PIT cannot be simply explained in terms of a S-O-R chain plus an aspecific excitatory process. At the very least, a third inhibitory process is present in the experimental data, waiting to be explained. We have suggested an hypothesis that can both explain how these three processes work and what is their function. We have linked specific PIT, general PIT and PIT inhibition to *three principles of goal-directed action*. The idea is that these three PIT processes represent the effect of conditioned Pavlovian stimuli on three aspects of the action evaluation that happens in *goal-directed* behavior. These three aspects are: *context* (an action directed to a goal needs the right context), *efficacy* (an action can have more or less chances to accomplish a certain goal) and *utility* (the consequences of an action can be more or less useful). According to this, specific PIT represents the use of cues that indicate higher chances of getting a certain reward. Data supporting the relationship between specific PIT and reward probabililities can be found in Trick et al. (2011) where those CS associated with higher chances of reward elicited stronger PIT. Explaining specific PIT in terms of probability would also agree with the fact that specific PIT is not influenced by devaluation procedures (Rescorla, 1994; Holland, 2004; Corbit et al., 2007): indeed the probability of getting a reward is independent of its value. Instead, general PIT affects utility of the action, its value, so it is subject to devaluation (Corbit et al., 2007). General PIT corresponds to the use by the agent of cues that indicate the presence of an additional reward in the immediate future and this motivates the animal to execute its actions with more vigor. Lastly, the inhibition of general PIT in some situations corresponds to the use of cues to understand which rewards are available in a given context.

We have then created a model of how this functional hypothesis could be translated into probabilistic computation. In particular, we have created a model drawing inspiration from Courville et al. (2005) where Pavlovian conditioning is explained in terms of a latent-cause generative model. By adding an action node and its links to food we have simulated instrumental learning, thus explaining the three PIT effects in terms of interactions between latent causes representing the contexts learned during Pavlovian conditioning and those learned through instrumental conditioning.

The model produces new predictions that might be tested in future empirical research and expand our knowledge about PIT. In particular, if specific PIT is an effect of “augmenting chances of success,” then instrumental actions that already have 100% chances of success should not be able to benefit from specific PIT (but they could still benefit from general PIT). The reinforcement schedules used to test PIT are non-continuous, so pressing the lever has not 100% chance of delivering food. It will be interesting to see if under a continuous reinforcement schedule this prediction will be confirmed or not. As for the principle of *context*, we might expect that in an experimental procedure where the two levers could be somewhat linked to the same context instead of “excluding each other,” we should observe less or no PIT inhibition. Indeed in the experiment of Corbit and Janak (2010) where rats were presented levers in an alternating fashion but within single sessions, results indicate a strong PIT effect in the *different* condition too, albeit somewhat less than the *same* condition (see Figure 5). A specific procedure focusing on the role of context might shed further light to confirm this part of the hypothesis. As for the *utility* effect, our hypothesis is not enough detailed to go beyond the fact that general PIT should be subject to devaluation, as already shown in the literature (Corbit et al., 2007). We do not know if the “additional reward” needs to be a different type of food compared to the instrumental action, or if a CS that signals the same food, but in a larger quantity, could be equally effective in producing general PIT.

In our attempt to provide an hypothesis capable of explaining all three PIT processes, we have focused on results achieved with the paradigm capable of detecting all of them (Corbit and Balleine, 2005; Corbit et al., 2007; Corbit and Balleine, 2011). However, those experiments involved multiple levers and a random-ratio schedule, thus evoking goal-directed behavior. This is why our hypothesis is proposed to be an explanation of how PIT processes affect goal-directed behavior. However, most of PIT experimental data has actually been produced using interval schedules (see Holmes et al., 2010 for a review). If those schedules have often evoked *habitual* responding, then a lot of data would fall outside the main focus of our hypothesis, which is limited to *goal-directed* behavior. That being said, we do have some suggestions on how PIT might work during habitual behavior. We have indeed suggested that specific PIT can only happen during *goal-directed* behavior as it pertains the chances of achieving a goal. During habitual behavior, there is no goal evaluation and thus we suggest that a CS paired with the same reward of the lever would produce general PIT instead. Then, some of the differences found in literature data could be explained by the use of different reinforcement schedules, leading to *habitual* vs. *goal-directed* behavior (e.g., Hall et al., 2001 vs. Blundell et al., 2001).

Beyond the contrast between Hall et al. (2001) and Blundell et al. (2001), we have another-contradiction in the literature which could be mostly resolved by differences of how PIT works under goal-directed vs. habitual behavior. We have mentioned above that Holland (2004) reported results that indicate that both specific and general PIT are insensitive to devaluation. This is in contrast with the results of Corbit et al. (2007) where only specific PIT is immune to devaluation. The results from Holland (2004) can be mostly reconciled by noting that he used an interval schedule, thus probably eliciting in many cases habitual behavior. In all those cases, the fact that general PIT was not affected by the devaluation procedure can be explained by the fact that under habitual behavior the baseline performance is not subject to devaluation either. In other words, we might suppose that under habitual behavior the devaluation process is “inactive” and thus it does not affect general PIT either. This would reconcile all results from the experiments of Holland (2004) except one: one of the experimental groups displayed infact a devaluation effect on baseline performance but not on the general PIT effect (Figure 4D in his article). That result would still conflict with Corbit et al. (2007)'s results, even after the additional assumption that general PIT might not be subject to devaluation under habitual behavior. The solution to this contradiction might lie on the devaluation procedure used. While Corbit et al. (2007) used satiety to devalue food rewards, Holland (2004) used an aversion procedure (pairings with LiCl): it might then be that the two devaluation procedures affect differently general PIT.

In our computational model, the analysis is limited to how the agent can make different inferences depending on the test conditions but it does not show how these inferences are transformed into action. We need to build a more complete model that can account on how these perceived *higher efficacy* or *additional rewards* are transformed into an higher instrumental performance. Moreover, the model is purely functional and does not yet shed light on the neural mechanisms underlying PIT. Neural models might be developed to this purpose. Future developments of the model and the hypothesis should also address conditions such as the use of drugs of abuse and chronic stress, which are known to have an effect on PIT (Wyvell and Berridge, 2001; Corbit and Janak, 2007; Morgado et al., 2012).

Despite the above mentioned limitations, we think our hypothesis can give a new perspective on PIT, a new framework on which to discuss, experiment, and advance our knowledge on PIT. Of particular importance is the investigation of *why* there is a PIT effect, i.e., its role in animal adaptation. We have done a first step in this direction by proposing that PIT relates to the ability of using signals in the environment to better evaluate the possibilites of action.

### Conflict of interest statement

The authors declare that the research was conducted in the absence of any commercial or financial relationships that could be construed as a potential conflict of interest.
